# Prevalence of Gingival Recession in the Nepalese Population Visiting Tertiary Care Hospital

**DOI:** 10.31729/jnma.9055

**Published:** 2025-06-30

**Authors:** Arjun Hari Rijal, Ruben Napit, Purnima Mahatara Chhetri, Sujata Pandey, Sandhya Poudel, Pratistha Ghimire, Bhojraj Adhikari, Simant Lamichhane, Manoj Humagain

**Affiliations:** 1 Department of Periodontology and Oral Implantology, Kathmandu University School of Medical Sciences- Dhulikhel Hospital, Dhulikhel, Kavrepalanchok, Nepal; 2 Dental Department, Methinkot Hospital, Bhakundebesi, Kavrepalanchok, Nepal; 3 Department of Oral and Maxillofacial Pathology, Kathmandu University School of Medical Sciences- Dhulikhel Hospital, Dhulikhel, Kavrepalanchok, Nepal

**Keywords:** *etiology*, *gingival recession*, *miller's classification*

## Abstract

**Introduction:**

Gingival recession is the exposure of root surface by an apical shift in the position of the gingiva. Exposure of the tooth surface leads to caries of the root surface, abrasion, erosion, sensitivity, esthetic concern and interproximal plaque accumulation. So, the objective of the present study was to find out the prevalence of recession in Nepalese population and its etiological factors.

**Methods:**

A descriptive cross-sectional study was conducted among 350 participants for a period of December 2023 to February 2024 at Department of Periodontology and Oral Implantology, Dhulikhel Hospital after obtaining ethical approval from Institutional Review Committee (Reference no: 229/23). Written informed consent was obtained and gingival recession and its associated factors were examined using well-formed proforma. Data were entered into Microsoft excel and descriptive statistics were reported.

**Results:**

Out of a total of 350 participants, the prevalence of gingival recession was found to be 170 (48.56%, 95% CI = 43.33-53.73%). Among the participants, 177 (50.57%) were male, with 86 (48.58%) having gingival recession, while 84 (48.55%) of the remaining female participants had gingival recession. In the 40-49 years age group, 62 (72.09%) individuals had gingival recession. Gingival recession was observed in 48 (64.87%) participants with poor oral hygiene, 31 (67.39%) using a hard toothbrush, 22 (61.12%) with a history of prosthodontic treatment, 7 (70%) with subgingival restorations, and 3 (100%) with papillary penetrating frenal attachment.

**Conclusions:**

The study reported a high prevalence of gingival recession among sample Nepali adult population compared to other studies and there were multiple etiologies associated with it.

## INTRODUCTION

Gingival recession is defined as location of marginal periodontal tissues apical to cementoenamel junction.^1^ The apical shift of gingiva leads to exposure of the root surface leading to subsequent problems associated with it.^2,3^ Exposure of the tooth surface also leads to sensitivity, caries of the root surface, abrasion, erosion, and interproximal plaque accumulation.^2-4,6^ The factor that contributes to recession includes inadequate brushing, destructive periodontal diseases, tooth malposition, bone loss, thin marginal tissue, tissue dehiscence, shallow vestibule, frenal pull, trauma from occlusion, orthodontic forces, smoking and sub gingival restorations.^3-5^ There is limited data regarding prevalence of gingival recession and its etiological factors in Nepal.

The prevalence of recession for population greater than 50-year-olds was found to be 99%, while in Nepal, the prevalence has been reported as 21.3% to 65.44%.^4-6^ So, this study aimed to find the prevalence of gingival recession and associated factors in patients visiting tertiary care hospital.

## METHODS

An observational cross-sectional study was conducted at Department of Periodontology and Oral Implantology, Dhulikhel Hospital from December 2023 to February 2024. Ethical approval was taken from Institutional Review Committee (IRC) of Kathmandu University School of Medical Sciences (KUSMS) (Reference number: 229/23).

A non-probability convenience sampling technique was used and the total sample size of 350 was calculated by utilizing following formula:

n =( Z^2^ × p × q)/e^2^

 = (1.96 × 0.65 × 0.35)/ (0.05)^2^

 = 349.59 = 350

Where,

Z = 1.96 at 95% Confidence Interval (CI)

p = prevalence of gingival recession, 0.65^5^

q = 1 -p

e = margin of error, 0.05.

The inclusion criteria included were patient above the age of 18 years, minimum of 24 natural teeth present excluding third molars, periodontal health or gingivitis while the third molars, root stumps, non-carious lesions in cervical area of multiple teeth where Cemento-enamel junction cannot be visualized, patientundermedication associatedwith gingival enlargement, systemic disease affecting periodontal tissue (bleeding disorder, diabetes mellitus) were excluded from the study.

The inclusion criteria were patient above the age of 18 years, having minimum of 24 natural teeth present excluding third molars, patients with either localized or generalised periodontitis. While the third molars, root stumps, non-carious lesions in cervical area of multiple teeth, the patients with unclear cementoenamel junction, under medication associated with gingival enlargement, systemic disease affecting periodontal tissue (bleeding disorder, diabetes mellitus) were excluded from the study.

Patients who met the inclusion criteria were invited to participate in the study and written consent was obtained before data collection. Participants were examined by two well-trained examiners and inter-examiner reliability was calculated use the Cronbach alpha value which was 0.87. The University of North Carolina - 15 (UNC-15) Periodontal Probe was be used to measure the linear measurement from the Cementoenamel junction to the margin of the gingiva. The recession was recorded according to P.D. Miller Jr.'s classification.^7^ Mid-buccal surface recession was measured and graded. In the presence of heavy band of calculus around CEJ, oral prophylaxis was done first and then measurements were recorded. Information regarding etiological factors ( gender, age in years: ≤19, 20-29, 30-39, 40-49, 50-59, ≥60 , frequency of tooth brushing: three times a day or more, two times a day, one times a day, duration of tooth brushing: one minute, two minutes, three minutes, four minutes, >four minutes, types of toothbrush: hard, medium, soft, types of dentifrice: powder, paste, brushing technique: horizontal, vertical, modified bass, fones, mixed technique, oral hygiene status: good, fair, poor, orthodontic treatment: done, not done, prosthodontic treatment done, not done, subgingival restoration: done, not done, smoking: yes, no, maxillary/mandibular frenal attachment were recorded in the structured proforma.

## MILLER'S CLASSIFICATION OF GINGIVAL RECESSION:

Class I: Marginal tissue recession without extending to the muco-gingival junction without loss of bone or soft tissue in the interdental areas.

Class II: Marginal tissue recession that extends to or beyond the muco-gingival junction without loss of bone or soft tissue in the interdental areas

Class III: Marginal tissue recession that extends to or beyond the muco-gingival junction in addition there is bone and/ or soft tissue loss in the interdental areas and/or mild malocclusion

Class IV: Marginal tissue recession that extends to or beyond the muco-gingival junction with severe bone and/or soft tissue loss in the interdental areas and/or severe tooth malposition

Data were entered in the Microsoft excel and descriptive analysis was done.

## RESULTS

Among total participants 177 (50.57%) were male and rest were female. Other demographic parameters are as shown (Supplementary File, Table 1).

Males have gingival recession prevalence of 86 (48.58%) and females have 84 (48.55%) prevalence. Out of total 350 participants, the prevalence of gingival recession was found to be 170 (48.56%). There was highest prevalence with poor oral hygiene 48 (64.87%), use of hard tooth brush 31(67.39%), prosthodontic treatment 22(61.12%), subgingival restoration 7(70%) and due to papillary penetrating frenal attachment 3 (100%). Similarly, 40-49 years age groups were mostly affected 62(72.09%) by gingival recession (Supplementary File, Table 2).

**Figure 1:**
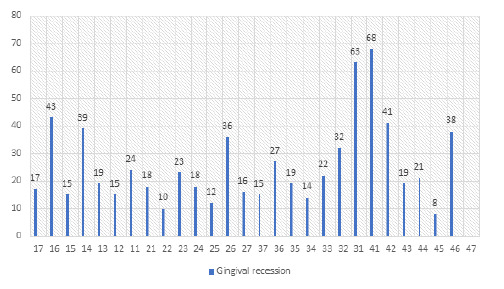
Prevalence of gingival recession according to tooth (n).

The gingival recession was more prevalent in 31 and 41 as 63 (17.99%) and 68 (19.42%) respectively (Table 1, Figure 1). Similarly, class III gingival recession was more prevalent in 31 and 41 as 30 (8.85%) and 30 (8.85%) respectively (Table 1, Figure 1). Most commonly affected tooth were lower incisors and least affected tooth was lower premolars (Figure 1).

**Table 1:** Prevalence of different parameters of gingival recession.

Tooth Number	Prevalence of gingival recession n(%)	Extent of gingival recession n (%)				Linear measurements of gingival recession (mm), n(%)		
		Class I	Class II	Class III	Class IV	≤3	4-5	>5
17	17 (4.85)	15(4.28	1(0.28)	1(0.28)	-	14(82.35)	3(17.64)	
16	43(12.80)	38(10.85)		5(1.42)		40(93.03)	1(2.33)	2(4.65)
15	15(4.28)	11(3.14)	1(0.28)	3(0.85)		15(100)		
14	39 (11.14)	35(9.99)	2(0.57)	2(0.57)		36(92.30)	1(2.56)	2(5.12)
13	19(5.42)	15(4.28)	2(0.57)	2(0.57)		17(89.48)	2(10.53)	2(10.53)
12	15(4.28)	12(3.42)		3(0.85)		15(100)		
11	25(6.85)	20(5.71)		5(1.42)		19(79.17)	3(12)	3(12)
21	17(5.14)	9(2.57)		6(1.71)	2(0.57)	12(70.59)	4(23.53)	1(5.88)
22	10(2.85)	7(1.99)		3(0.28)		7(70.00)	3(30.00)	
23	23(6.57)	17(4.85)	1(0.28)	5(1.42)		21(91.30)	1(4.34)	1(1.34)
24	18(5.14)	15(4.28)		3(0.85)		15(83.34)	2(11.12)	1(5.56)
25	12(3.42)	9(2.57)	1(0.28)	2(0.57)		12(100)		
26	36(10.28)	29(8.28)	1(0.28)	5(1.42)		33(91.67)	3(8.33)	
27	16(4.57)	15(4.28)	1(0.28)			14(87.5)	2(12.5)	
37	15(4.28)	12(3.42)		3(0.85)		15(100)		
36	27(7.71)	22(6.28)		5(1.42)		21(77.78)	6(22.22)	
35	19(5.42)	16(4.57)		2(0.57)		19(100)		
34	14(3.99)	12(3.42)		2(0.57)		11(78.58)	2(14.29)	1(7.13)
33	22(6.28)	10(2.85)	1(0.28)	11(3.42)		16(72.73)	5(22.73)	1(4.54)
32	32(9.14)	13(3.42)	4(0.98)	15(4.28)		24(75.00)	6(18.75)	2(6.25)
31	63(17.99)	30(8.85)	3(0.85)	30(8.85)		43(68.25)	7(11.12)	13(20.63)
41	68(19.42)	38(11.14)		30(8.85)		52(76.47)	14(60.29)	2(2.94)
42	41(11.71)	15(4.28)	1(0.28)	20(5.71)		26(63.42)	10(224.39)	5(12.19)
43	19(5.42)	10(2.85)	1(0.28)	8(2.28)		14(73.68)	5(26.32)	
44	21(5.99)	18(5.14)		3(0.85)		16(76.19)	5(23.81)	
45	8(2.280	6(1.71)		2(0.57)		8(100)		
46	38(10.85)	34(9.71)		4(1.14)		34(89.47)	4(10.13)	
47	16(4.57)	15(4.28)		1(0.28)		16(100)		

## DISCUSSION

Out of 350 participants in the study, the prevalence of gingival recession was found to be 170. Miller's classification of gingival recession, introduced in 1985, remains one of the most widely used systems for categorizing gingival recession in clinical and research settings.^7^ Despite being developed over three decades ago, it continues to play a central role in periodontal research and practice. The primary reason for its continued use is its simplicity and clinical relevance. Miller's classification divides gingival recession into four classes (I-IV) based on the extent of soft tissue loss and the potential for root coverage. This classification provides a quick and practical way to assess the severity of recession and predict the outcome of root coverage procedures. Because it is easy to apply and understand, it is favored by clinicians, educators, and researchers alike. In current research, Miller's classification is frequently used to standardize patient selection, categorize defects, and compare treatment outcomes across different studies. Its long-standing use also allows comparison with historical data, which is important for evaluating long-term trends in periodontal health and treatment success. However, despite its popularity, the system is not without limitations. It does not consider the three-dimensional aspects of tissue loss, interdental clinical attachment levels, or the presence of multiple recession defects. Newer classifications, such as the Cairo classification, aim to overcome these limitations by including more precise diagnostic criteria. Still, due to four categories: anatomical, pathological, traumatic or its simplicity, ease of use, and historical acceptance, Miller's classification remains a benchmark in periodontal research. Researchers continue to use it, either alone or alongside newer systems, to maintain consistency and clarity in reporting. In summary, although it is the oldest classification, Miller's system continues to hold value in current research due to its practicality and widespread recognition.

In the present study, participants meeting inclusion criteria were included from the Department of Periodontology and Oral Implantology, KUSMS-DH. Gingival recession being one of the most prevalent periodontal condition affecting the oral health related quality of life. So, we tried to find out the overall prevalence of gingival recession by examining the entire dentition in contrast to other studies conducted in Nepal which were limited to either orthodontic patient6 or to the lower anterior only^8^.

Despite common observation in adults; the prevalence, extension and degree of severity of gingival recession present considerable differences among various study populations.^8-10^ Prevalence indicates number of cases or occurrences of gingival recession; extension corresponds to the number of teeth affected by gingival recession; and severity signifies the total root surface exposed by the gingival recession, i.e. the linear apico-coronal height of the gingival recession. The overall prevalence of gingival recession in the current study was 170 (48.56%). Similar findings were found in the other studies done by Shakya et al.^8^ 121(46.54%), Manchala et al.^9^ , Rios et al.^10^ 3496 (51.10%), Fragkioudakis et al.^2^ 56(53.80%), Romano et al.^11^ 57.20% whereas other studies done by Romandini et al.^12^ 91.6%, Strauss et al.^13^ 90.9%, Kholti et al. 354(88.5%) found higher prevalence of gingival recession. The higher prevalence of the gingival recession may be due to larger number of sample size included in the study, data extracted from the national survey. Also, the less prevalence in our study may be due to hospital-based cross-sectional survey conducted for limited period of time.

In the present study, the prevalence of gingival recession was higher in mandibular central and lateral incisors. Similar findings were observed in several studies conducted around the globe.^8,9,14,15^ Whereas other studies conducted by Frakioudakis et al.^2^, Vignoletti et al.^16^, Slutzkey et al.^17^ reported higher prevalence in maxillary premolar and molars. Similarly, prevalence of gingival recession was less in lower left premolar in the present study. In contrast, it was higher according to the results shown by Frakioudakis et al.^2^. The higher prevalence of gingival recession in the mandibular anterior region may be due to high frenal attachment, inadequate vestibular depth, incisal crowding as compared to maxillary incisors.

In the current study, female 84(48.58%) had slightly lower gingival recession as compared to male 86(48.56%). Similar trends can be seen in the previous studies conducted by Shakya et el.^8^, Slutzkey et al.^17^, Toker et al.^3^.

Different etiological factors of gingival recessions were identified in the literature.^5-7^ They could be categorized into iatrogenic factors. These factors could be interrelated leading to the development of gingival recession defects. Hence, in most cases, we could identify more than an etiological factor. Therefore, the presence of one of these factors may increase the potential risk of developing gingival recessions. Different risk indicators were reported in the literature in different populations. Among these risk indicators, age has been mostly associated with the presence of gingival recession.

We divided age into six groups as ≤19, 20-29, 30-39, 40-49, 50-59 and ≥60 years in the current study. The prevalence of gingival recession seemed to be increasing with increasing age with 1(20.00%) in ≤19 years, 33(34.02%) in 20-29 years, 35(56.45) in 30-39 years, 37(47.43) in 40-49 years, 62(72.09) in 50-59 years, 2(9.09) in >60 years group. The maximum gingival recession was found in 50-59 years age group and the minimum in the ≤19 years age group. The was also increasing trend up to 50-59 years age group followed by drop in the prevalence in the ≥60 years age group may be due to very few participants in that particular age group. The higher prevalence of gingival recession with increasing age may be attributed to the cumulative impact of aging, periodontal disease, and prolonged exposure to factors that contribute to gingival recession.

In the present study, we examined about the effects of toothbrush frequency, duration of toothbrushing, types of toothbrush, types of dentifrices and technique of toothbrushing on gingival recession. The results showed the patient who brushes one times a day for more than four minutes with hard tooth brush and toothpaste had tendency to develop gingival recession. Similar findings was illustrated by Abdulhamed in their study.^18^

Similarly, one of the important factors that have impact on initiation and progression of gingival recession is the status of oral hygiene. In the present, Oral Hygiene Index- Simplified (OHI-S) was used to find out the oral hygiene status of the participants. In patients with poor oral hygiene the gingival recession was more prevalent 64.87%. There was association between the presence of calculus and prevalence of gingival recession in a study conducted by Sushin et al.^19^ in Brazilian population.

Smoking is also considered as the one of the major risk factors for the development of loss of attachment. Progression of localized or generalized form of gingival recession is usually associated with the presence of subgingival calculus and smoking habit.^3^ In the present study, most of the participants 240(68.58%) were non-smoker. Whereas the prevalence of gingival recession is higher in the participants with smoking habits 68/110(61.81%). Similar result was shown by Manchala et al. in their study.^9^

Though the present study collected the prevalence, extent, severity and factors associated with gingival recession, it has certain limitations. It is cross-sectional study conducted for short period of time. So, the findings couldn't provide data of longer duration and cause/effect relations also couldn't be established. Its only snapshot of gingival recession for a brief period of time. This study was conducted in a university teaching hospital with limited sample size, so it can't be generalised in the larger population.

To overcome the limitation of the present study, we recommend to conduct a longitudinal study with large sample for longer period of time.

## CONCLUSIONS

Gingival recession is one of the more prevalent periodontal condition in the studied population attended in the tertiary care hospital and brushing characteristics, oral hygiene status, smoking, etc. are the associated risk factors for gingival recession.
